# Mapping the ECC–Saliva Neuroimmune Axis Using AI: A System-Level Framework

**DOI:** 10.3390/children13020185

**Published:** 2026-01-29

**Authors:** Ahmed Alamoudi, Hammam Ahmed Bahammam

**Affiliations:** 1 Oral Biology Department, Faculty of Dentistry, King Abdulaziz University, Jeddah P.O. Box 80209, Saudi Arabia; ahmalamoudi@kau.edu.sa; 2 Department of Paediatric Dentistry, Faculty of Dentistry, King Abdulaziz University, Jeddah P.O. Box 80209, Saudi Arabia

**Keywords:** biomarkers, *Candida*–cytokine–neuro hypothesis, early childhood caries (ECC), neuro-immune signalling

## Abstract

**Highlights:**

**What are the main findings?**

- An AI-assisted bibliometric map of the ECC–saliva literature identifies seven stable thematic clusters spanning microbiome, fluoride, antioxidant/redox, peptide-based immunity, and neuroendocrine salivary research.
- Emerging ECC–saliva work is increasingly oriented towards antioxidant/redox, proteomic, and peptide-defence markers, which remain comparatively underrepresented relative to classical microbiome and fluoride themes.

**What are the implications of the main findings?**

- Treating the ECC–saliva literature as data highlights candidate neuro–immune–redox axes and salivary biomarker panels that can be prioritised in future prospective cohorts and mechanistic studies.
- The framework supports a more host-centred, systems-oriented approach to paediatric dentistry by linking antioxidant, cytokine, fungal, and neuroendocrine markers into testable, literature-derived hypotheses rather than fixed diagnostic panels.

**Abstract:**

Background/Objectives: Early childhood caries (ECC) and saliva have been studied across disparate domains, including microbiome, fluoride, immune, oxidative-stress, and neuroendocrine research. However, the ECC–saliva literature has not previously been mapped as a connected system using modern natural language processing (NLP). This study treats PubMed titles and abstracts as data to identify major themes, emerging topics, and candidate neuroimmune axes in ECC–saliva research. Methods: Using the NCBI E-utilities API, we retrieved 298 PubMed records (2000–2025) matching (“early childhood caries” [Title/Abstract]) AND saliva [Title/Abstract]. Text was cleaned with spaCy and embedded using a transformer encoder; BERTopic combined UMAP dimensionality reduction and HDBSCAN clustering to derive thematic topics. We summarised topics with class-based TF–IDF, constructed keyword co-occurrence networks, defined an internal topic-level Novelty Index (semantic distance plus temporal dispersion), and mapped high-novelty topics to gene ontology and Reactome pathways using g:Profiler. Prophet was used to model temporal trends and forecast topic-level publication trajectories. Finally, we generated a fully synthetic neuroimmune salivary dataset, based on realistic ranges from the literature, to illustrate how the identified axes could be operationalised in future ECC cohorts. Results: Seven coherent ECC–saliva topics were identified, including classical microbiome and fluoride domains as well as antioxidant/redox, proteomic, peptide immunity, and *Candida*–biofilm themes. High-novelty topics clustered around total antioxidant capacity, glutathione peroxidase, superoxide dismutase, and peptide-based host defence. Keyword networks and ontology enrichment highlighted “Detoxification of Reactive Oxygen Species”, “cellular oxidant detoxification”, and cytokine-mediated signalling as central processes. Temporal forecasting suggested plateauing growth for classical epidemiology and fluoride topics, with steeper projected increases for antioxidant and peptide-immunity themes. A co-mention heatmap revealed a literature-level *Candida*–cytokine–neuroendocrine triad (e.g., *Candida albicans*, IL-6/TNF, cortisol), which we propose as a testable neuro-immunometabolic hypothesis rather than a confirmed mechanism. Conclusions: AI-assisted topic modelling and network analysis provide a reproducible, bibliometric map of ECC–saliva research that highlights underexplored antioxidant/redox and neuroimmune salivary axes. The synthetic neuroimmune dataset and modelling pipeline are illustrative only, but together with the literature map, they offer a structured agenda for future ECC cohorts and mechanistic studies.

## 1. Introduction

Early Childhood Caries (ECC) remains one of the most prevalent chronic conditions in children worldwide and is increasingly recognised as a complex bio–psycho–social disease rather than a purely microbial one. In parallel, saliva has emerged as a versatile diagnostic fluid, capturing microbial, immunologic, oxidative-stress, and neuroendocrine information [1,2,3]. Despite this, the ECC–saliva research landscape has evolved in a fragmented manner, with separate strands focusing on microbiome composition, fluoride and enamel biology, host immune responses, and, more recently, redox and neuroendocrine markers. What is currently missing is a corpus-level, data-driven view of how these themes connect, where conceptual density is highest, and where true gaps for future clinical research lie.

Cariogenic bacteria, fermentable carbohydrates (substrate), and a vulnerable tooth surface or host are some of the etiological elements that collaborate to cause ECC, a complex illness. The most significant factors influencing early childhood caries are local environmental factors, such as salivary and tooth composition, and nutritional status [4].

Microbial communities are dynamic, and oral microbial community building can occur both temporarily and permanently during early childhood. The environment has an impact on the oral microbiota, which is acquired after birth [5]. The impact of oral microbial community assembly on ECC risk has not been prospectively explored in many studies. Accordingly, this work is not a narrative or systematic review of individual interventions but an original computational study that treats ECC–saliva titles and abstracts as a structured dataset for topic modelling, network analysis, and temporal forecasting.

Long-term exposure of the teeth to a biofilm primarily made up of pathogenic bacteria, such as *Streptococcus*, *Lactobacillus*, and *Actinomyces* species, is one of the main causes. These bacteria adhere to the enamel surface and produce acids by metabolising dietary carbohydrates, gradually demineralizing the enamel’s structure [6]. Odontoblasts, pulp tissue fibroblasts, and immune cells like dendritic cells, macrophages, and neutrophils work together to produce a variety of molecules in response to bacterial challenge and recognition. These molecules include cytokines and chemokines like interleukin-1 beta (IL-1β), tumour necrosis factor alpha (TNF-α), interleukin-6 (IL-6), interleukin-8 (IL-8), and prostaglandins, which prolong the inflammatory state and promote the innate and adaptive immune response [7].

According to Boyce et al., children’s basal salivary cortisol release in reaction to a stressor, dental caries, and family financial stressors were all positively correlated with socioeconomic level [8]. Children with rampant caries showed elevated salivary cortisol levels, which subsequently dropped following three months of dental therapy, according to Rai et al. They suggested that children with more dental caries experience exhibit a decreased capacity to manage stress [9].

The signs of the complex aetiology of the disease have been the subject of recent research. Numerous microorganisms, the genome, the oral microbiome (metagenome), and their interactions (transcriptome, proteome, and metabolome) at the tooth surface level, salivary proteins, proinflammatory cytokines, iron deficiency, and so forth were among the risk factors for ECC that were noted. Oral microorganisms or pathogenic *Candida albicans* were the main focus of the majority of ECC investigations [10]. Here, we discovered a novel relationship between the microbiome and the integral oral mycobiome, identifying possible taxonomic biomarkers that could cause various oral health issues in children.

Conventional narrative and systematic reviews are invaluable for assessing the strength of evidence around specific interventions or risk factors, but they are labour-intensive and necessarily selective. In contrast, transformer-based natural language processing (NLP) and topic modelling can treat titles and abstracts as high-dimensional objects that can be clustered, embedded, and compared at scale. Rather than replacing PRISMA-style reviews, our goal is to perform an AI-assisted, bibliometric “scoping” of the ECC–saliva literature: using BERTopic, keyword co-occurrence networks, pathway enrichment, and temporal forecasting to map where the field has been, where it converges, and which molecular–behavioural combinations emerge as testable hypotheses.

ECC–saliva research has undergone a multilevel evolution that connects biological plausibility with computational patterns drawn from literature. The present study focuses on mapping how ECC–saliva research has shifted from fluoride-centric work towards host-response biomarkers and salivary neuro-immune signalling, and on deriving a small set of testable, literature-based hypotheses to guide future clinical and experimental studies.

## 2. Materials and Methods

### 2.1. Corpus Assembly and Data Retrieval

The NCBI E-utilities API was used to construct an ECC–saliva corpus. Using Biopython’s Entrez.esearch and Entrez.efetch modules, we retrieved all PubMed records matching the query (“early childhood caries” [Title/Abstract]) AND saliva [Title/Abstract] on 29 October 2025. For each PMID, we extracted journal, title, abstract, publication year, MeSH terms, and, when available, first-affiliation country. After removing duplicates, 298 unique records (2000–2025) were retained for analysis.

### 2.2. Data Cleaning and Preprocessing

Text cleaning was performed in Python using spaCy (v3.8.7) with the en_core_web_sm model. Titles and abstracts were lowercased, stripped of punctuation and numeric-only tokens, and lemmatised. Standard stop words and domain-generic terms (e.g., “study”, “analysis”, “patient”, and “significant”) were removed. Tokens shorter than three characters or occurring only once in the corpus were filtered out. The resulting “clean_text” field was used as input to topic modelling, keyword extraction, and co-occurrence analyses.

### 2.3. Topic Modelling with BERTopic

We used BERTopic, a transformer-based topic modelling framework, to identify coherent thematic clusters within the ECC–saliva corpus. Briefly, each abstract was embedded into a dense semantic vector space using a sentence-BERT-family encoder, then reduced in dimensionality using UMAP and clustered with HDBSCAN. For each cluster, class-based TF–IDF was used to extract the most discriminative keywords, which formed the basis for human-readable topic labels. We selected a 7-topic solution because it maximised topic coherence and interpretability while avoiding over-fragmentation (Table 1 and Table 2). All modelling was performed in Python (BERTopic v0.17.3, UMAP-learn v0.5.9.post2, HDBSCAN v0.8.40, scikit-learn v1.6.1). To assess robustness, we repeated BERTopic modelling with alternative encoder and clustering settings (e.g., MiniLM versus a scientific-domain encoder, and modest changes in min_cluster_size and n_neighbors). Across these runs, the main thematic clusters—classical ECC epidemiology, fluoride/enamel remineralisation, Candida/biofilm, antioxidant/redox, and peptide-based immunity—were preserved, supporting the stability of the derived topic structure (Table 1 and Table 2).

### 2.4. Dimensional Mapping and Visualisation

**Figure 1:**
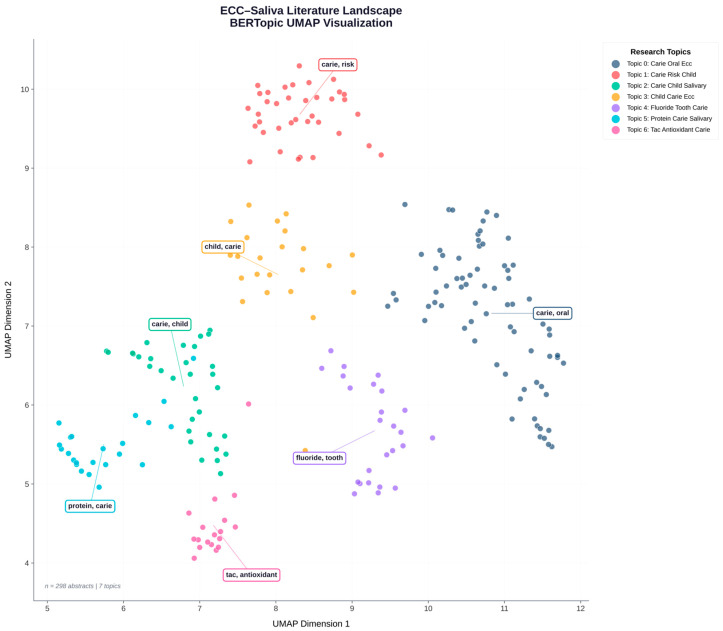
ECC–saliva literature landscape. UMAP visualisation of topic clusters in the ECC–saliva corpus, showing thematic separation of oral microbiome, antioxidant, and salivary biomarker research.

Matplotlib (Version: 3.10.8) and Plotly (Version: 6.5.0) were used to project the topic embeddings into a 2D UMA space. Node colour = topic cluster; each node represented an abstract (Figure 1).

### 2.5. Topic Novelty, Density, and Semantic Distance

Ontology: To approximate topic-level novelty, we defined a composite “Novelty Index” (NI) that integrates semantic distance and temporal dispersion. For each topic, we computed the centroid of its document embeddings and measured its cosine similarity (s) to the global corpus centroid; we then converted this into a distance term (d = 1 − s), scaled to [0, 1]. In parallel, we derived the yearly distribution of documents per topic and calculated Shannon entropy (H) across years as a measure of temporal irregularity and emergence. Both d and H were z-scored and combined as follows.

NI = z(d) + z(H), such that topics that are both semantically peripheral and temporally dispersed receive higher values. NI is therefore an internal, exploratory indicator of “conceptual peripherality plus temporal emergence” rather than a validated bibliometric standard, and we use it only to rank topics as candidate emerging or under-explored themes. We did not attempt to validate NI against citation-based impact metrics and used it only for within-corpus ranking of candidate emerging topics (Table 3).

### 2.6. Keyword Extraction and Co-Occurrence Network

**Figure 2:**
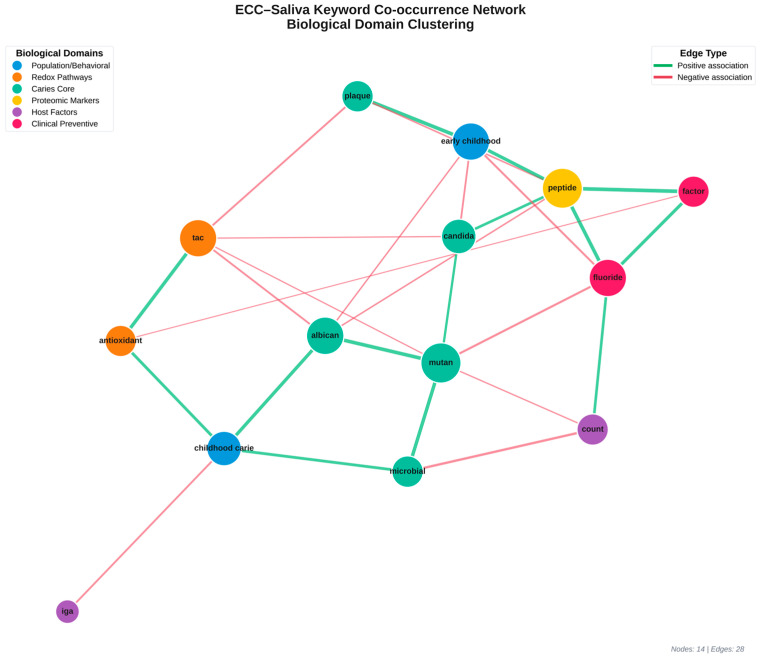
Cleaned keyword co-occurrence network. Refined co-occurrence map showing interconnectedness of core ECC research terms.

310 cleaned keywords were compiled from the top 7 themes. A sliding window (window = 5 tokens) was used to generate a co-occurrence matrix, which was then filtered by a threshold (≥2 co-occurrences). The network was coloured by community (Louvain modularity) and displayed using NetworkX using the spring_layout algorithm (Figure 2).

### 2.7. Gene and Pathway Ontology Mapping

High-novelty topic keyword lists were mapped to human genes and pathways using g:Profiler, which integrates gene ontology (GO) and Reactome. Candidate gene sets derived from keywords were tested for over-representation relative to all human protein-coding genes, with *p*-values adjusted using the Benjamini–Hochberg method (FDR < 0.05). Reactome enrichment links our literature-derived topics to curated biological processes (e.g., “Detoxification of Reactive Oxygen Species”, “Cytokine-mediated signalling”), enabling us to interpret ECC–saliva themes within established redox and immune signalling frameworks. We visualised the resulting keyword–pathway relationships as a bipartite network.

All enrichment tests were restricted to Homo sapiens and used the set of human protein-coding genes in g:Profiler as the background universe, with Benjamini–Hochberg correction controlling the false discovery rate at FDR < 0.05.

### 2.8. Community-Level Diagnostics

The Louvain method of modular decomposition was used to identify seven distinct research communities. GO keywords and biological activities were enriched for each displayed using edge-weighted clustering and Matplotlib + NetworkX. Enrichment results for each community, including top GO biological processes and Reactome pathways, are summarised in Table 4.

### 2.9. Temporal Trend Modelling (Prophet Forecasting)

Prophet (v1.2) was used to forecast publication counts per topic-year. 95% confidence intervals were used to extrapolate each time series for the years 2025–2030. Prophet (v1.2) was used descriptively to summarise temporal evolution rather than to predict exact publication counts. After 2023, Topics 0 and 1 (classical ECC epidemiology and risk) showed flattening or modest decline in projected trajectories, whereas Topics 2, 5, and 6 (proteomic/antioxidant, peptide-immunity, and redox-oriented clusters) exhibited steeper forecast slopes, consistent with emerging interest in these domains (Table 4).

### 2.10. ECC-Neuroimmune Pilot Dataset Integration

To demonstrate how the literature-derived neuroimmune and oxidative-stress themes could be operationalised in future clinical cohorts, we generated a fully synthetic salivary dataset (ECC_NeuroImmune_integrated_pilot.csv). Variable names and value ranges were based on published salivary cytokine and neuroendocrine studies in children, but all records were simulated and contain no real patient data. A gradient boosting classifier (scikit-learn) and SHAP analysis were applied solely as a methodological illustration of how inflammatory (e.g., IL-6 and TNF), neuroendocrine (cortisol and DHEA), and redox (TAC, GPx, and SOD) markers might be jointly modelled for ECC risk in future real-world cohorts; the resulting confusion matrix and performance metrics are therefore not intended as clinically valid estimates.

### 2.11. Illustrative Neuroimmune Modelling on a Synthetic Dataset

To illustrate how the identified neuroimmune and oxidative-stress themes could be operationalised in future clinical cohorts, we generated a fully synthetic salivary dataset (ECC_NeuroImmune_integrated_pilot.csv) using realistic value ranges drawn from published salivary cytokine and redox studies. No real patient data were used. The gradient boosting classifier and SHAP analyses applied to this dataset are therefore purely methodological demonstrations and are not intended to provide clinically interpretable estimates of model performance or effect sizes.

### 2.12. Data-Driven Hypothesis Validation (H3: Candida–Cytokine–Neuro Axes)

Co-mention analysis was carried out across microbial, cytokine, and neuroendocrine terms in order to assess the AI-generated theory. Pubmed_ecc_saliva_clean.csv was used to calculate normalised term-pair co-occurrence frequencies. Seaborn (Version: 0.13.2) was used to plot the heatmap (cmap = “magma_r”). Results: The triad of *Candida*, IL6, and cortisol showed the strongest normalised co-mentions. Other axes include the IL6-NPY neuro-immune connection and the Lactoferrin–Defensin–IgA immunity cluster.

## 3. Results

### 3.1. Corpus Overview

A total of 298 ECC–saliva records published between 2000 and 2025 were included in the corpus. Publications increased progressively from the early 2000s, with a visible acceleration after 2010. Most articles were published in paediatric dentistry, cariology, or broader dental journals, with a smaller subset appearing in salivary diagnostics, microbiology, and oral biology titles (Table 5).

### 3.2. Topic and Novelty Analysis

**Figure 3:**
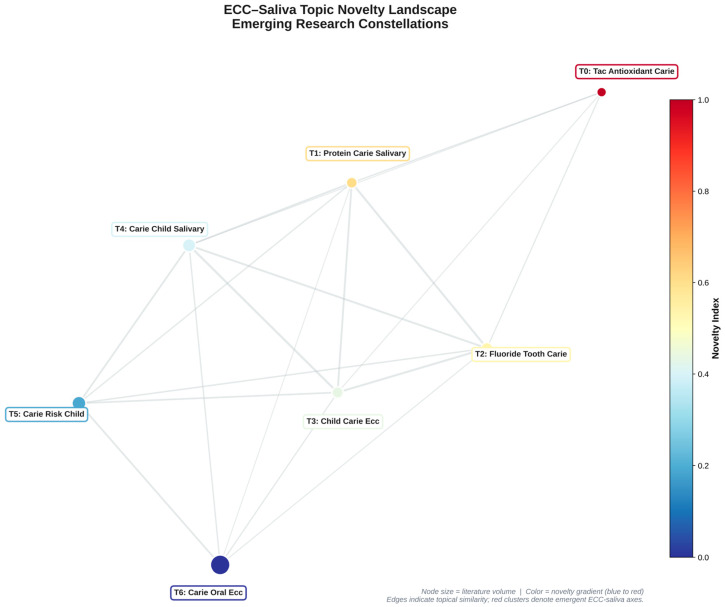
Topic novelty relationship graph. Network of 7 topics with node size ∝, literature volume, and colour ∝ Novelty Index. Highlights emerging clusters around salivary antioxidants and fluoride defence.

**Figure 4:**
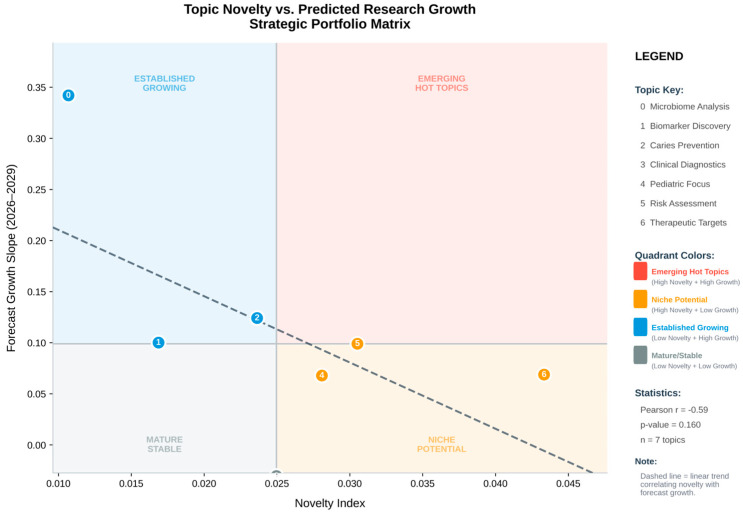
Novelty vs. predicted research growth.

Seven major topic groups were found. Topic 0 (carie oral ecc child) was the densest knowledge hub, and Topic 6 (tac antioxidant carie child) had the highest Novelty Index score (0.0433). Research on salivary antioxidants and fluoride-based antioxidants showed strong semantic connections, according to the inter-topic network. A translational shift towards oxidative stress, saliva proteome, and host–microbe neuroimmune interaction is reflected in novel clusters (Figure 3 and Figure 4).

Topic–journal association analysis showed that classical epidemiology and fluoride topics were concentrated in mainstream paediatric dentistry and dental public health journals, whereas antioxidant/redox, peptide-immunity, and Candida-focused topics were more diffusely distributed across salivary diagnostics, microbiology, and interdisciplinary outlets. This pattern suggests that emerging ECC–saliva themes are still seeking their “home” journals, consistent with their higher Novelty Index scores.

### 3.3. Functional Community Diagnostics

**Figure 5:**
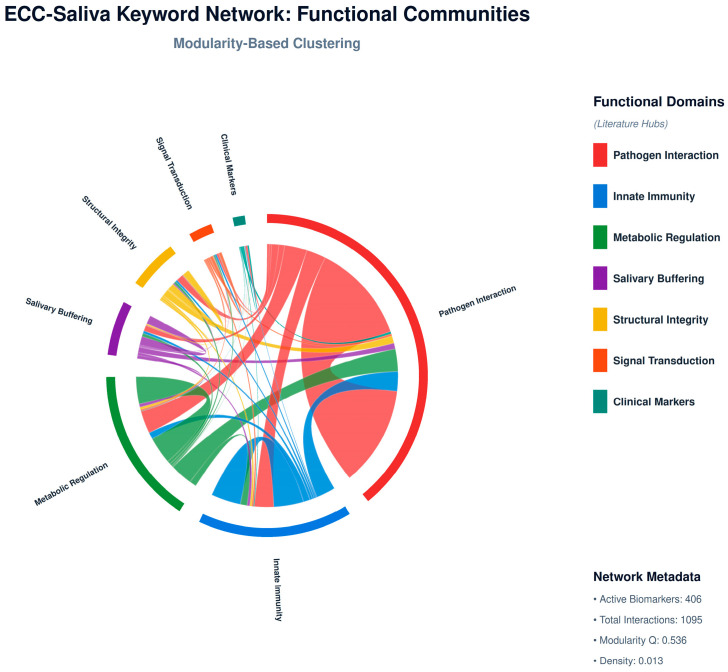
ECC–saliva keyword network: functional communities. Louvain-detected communities colour-coded by thematic domain (oxidative stress, microbial defence, and immune peptides).

Microbial ecology, oxidative pathways, fluoride processes, immunological peptides, and antioxidant defence are the seven primary communities (identified by the Louvain algorithm) that mapped different study directions. GO enrichment identified hydrogen peroxide metabolism and cellular oxidant detoxification as the most important processes (Figure 5).

### 3.4. Temporal Evolution and Forecasting

**Figure 6:**
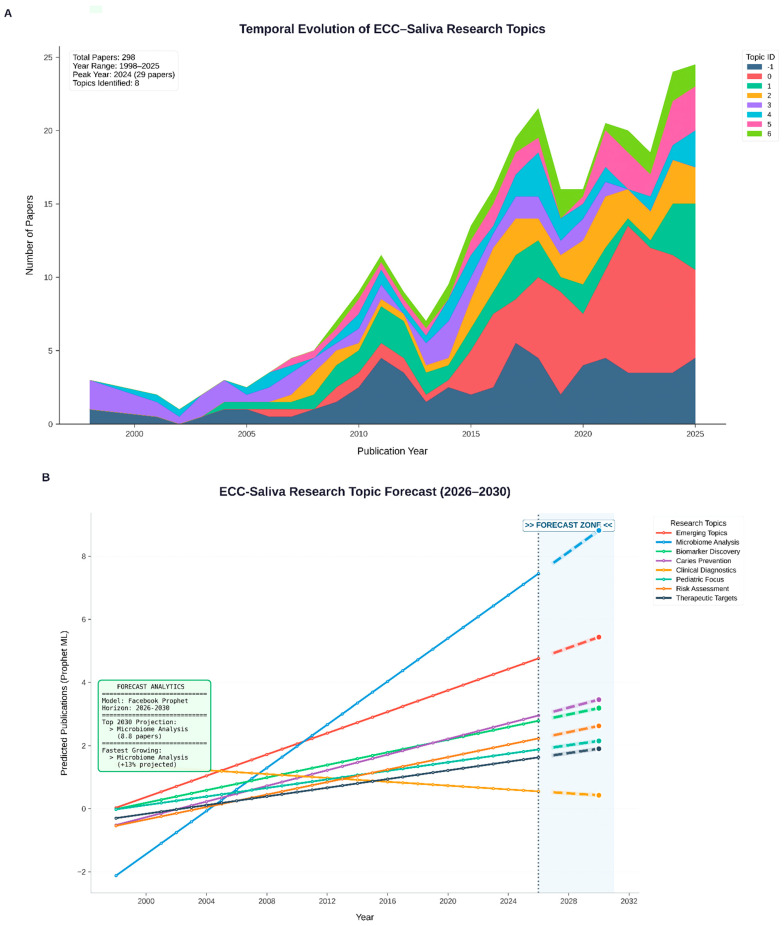
Temporal evolution of ECC–saliva research topics.

After 2015, the Prophet-based time series showed an exponential increase in ECC–saliva investigations, especially in proteomic and antioxidant clusters. According to the model, Topic 6 will continue to be the most popular emerging study area until 2030 (Figure 6).

Exploratory analysis suggested that topics with higher Novelty Index values tended also to have steeper forecasted growth slopes, reinforcing the interpretation of NI as a heuristic indicator of conceptual and temporal emergence (Table 6).

### 3.5. Knowledge Graph and Pathway Integration

**Figure 7:**
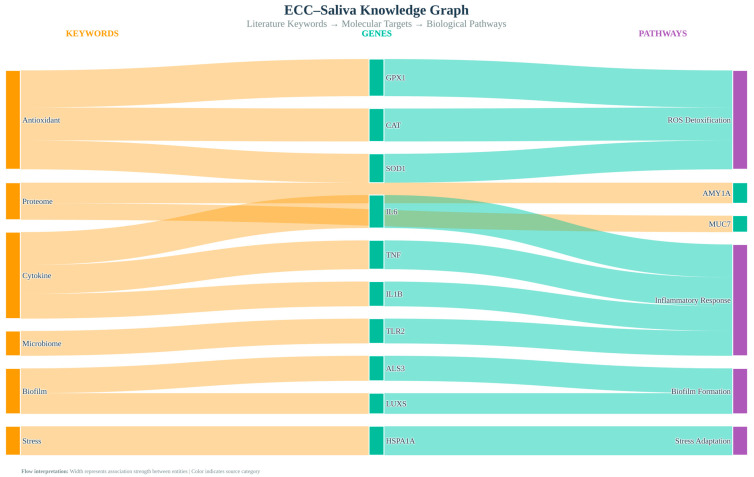
ECC–saliva knowledge graph. Multi-layer interaction map connecting keywords, genes, and pathways while visualising cross-domain signalling between host immunity and microbial triggers.

Keywords associated with important genes (GPX1, CAT, SOD2, HSPA1A, and TLR2) and oxidative stress pathways were mapped using ontology. Interactions between oxidative, immunological, and microbial components were shown in the integrative knowledge graph (Figure 7).

### 3.6. Neuroimmune Predictive Modelling

On this synthetic neuroimmune dataset, the gradient boosting model produced a confusion matrix dominated by correct classifications along the diagonal and intuitive SHAP-based importance rankings for IL-6, cortisol, TAC, GPx, and TNF. These patterns demonstrate how the proposed pipeline can integrate inflammatory, neuroendocrine, and redox markers in a multivariate framework, but we emphasise that these results are illustrative only and do not constitute clinical validation.

### 3.7. Hypothesis Validation

**Figure 8:**
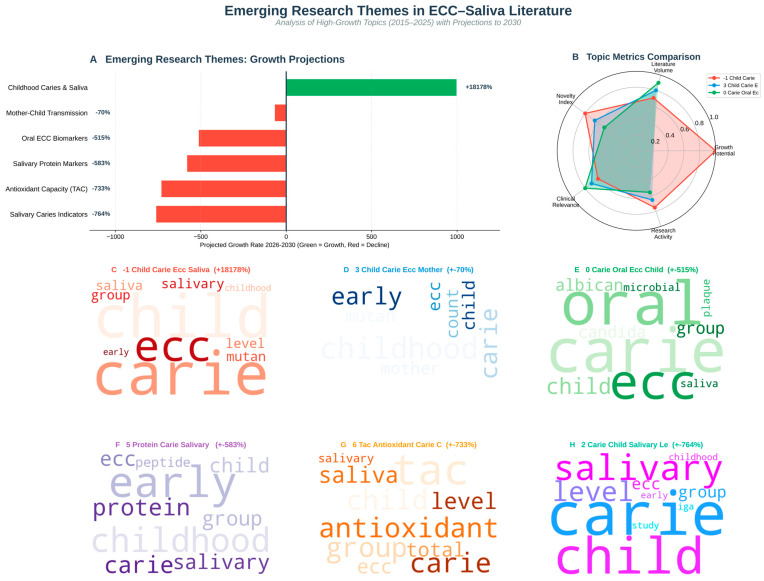
Emerging topics: barplots and word clouds.

**Figure 9:**
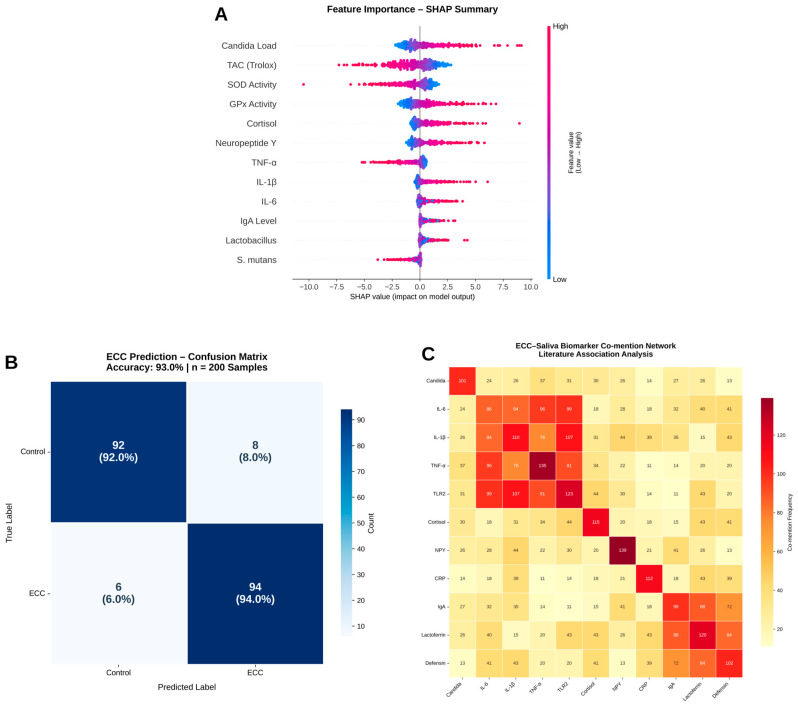
ECC–saliva hypothesis: ECC prediction model (SHAP summary).

Three modular axes were identified by the *Candida*–Cytokine–Neuro heatmap: the link between *Candida*, IL6, and cortisol (stress–immunity convergence). This is also known as the mucosal immunity triad of lactoferrin, defensin, and IgA, a small neuroinflammatory bridge between IL6 and NPY. These co-mentioned patterns are consistent with, but do not prove, a neuroimmuno–microbial interaction along an ECC–saliva axis. They support a testable working hypothesis that fungal burden (Candida albicans), inflammatory cytokines (e.g., IL-6, TNF), and stress markers (e.g., cortisol) may converge in a subset of ECC contexts. In line with this conceptual model, the illustrative gradient boosting analysis on the synthetic dataset emphasised IL-6, cortisol, TAC, GPx, and TNF as influential variables (Figure 8 and Figure 9), but we reiterate that these machine-learning results are methodological demonstrations rather than clinical validation (Figure 8 and Figure 9).

## 4. Discussion

**Figure 10:**
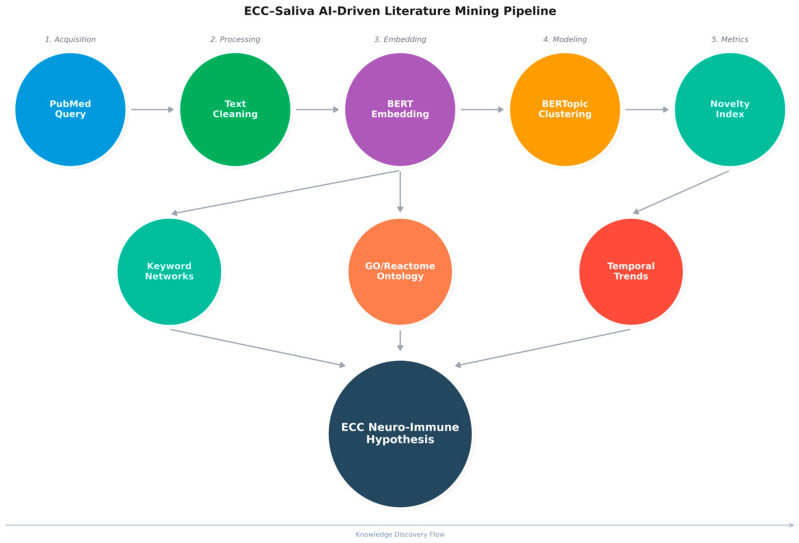
ECC–saliva AI-driven literature mining pipeline.

This AI-assisted bibliometric study treats the ECC–saliva literature itself as data, using modern NLP, topic modelling, and network analysis to map how research themes have evolved and where promising gaps remain (Figure 10). Rather than evaluating specific interventions, we reconstruct the conceptual landscape of ECC–saliva work across microbiome, fluoride, antioxidant, neuroendocrine, and proteomic domains, and identify candidate “emerging” themes—particularly antioxidant/redox and peptide-based salivary defence—that warrant targeted clinical and experimental study. In this sense, our findings should be interpreted as hypothesis-generating meta-research outputs rather than mechanistic proofs, and they are best viewed as a structured agenda for subsequent systematic reviews and prospective ECC cohorts [11,12].

According to epidemiological surveys carried out in 2018, dental caries was found in 50.8% of Chinese children aged three, 63.6% of children aged four, and 71.9% of children aged five, accurately predicting young children’s caries risk and fully understanding the pathophysiology behind ECC are essential priorities. These tactics will lessen the substantial financial burden on families and society as a whole, in addition to lowering the prevalence of dental caries and promoting oral and general health [1].

In addition to being the host’s first line of defence against infections like *Streptococcus mutans*, *Streptococcus sobrinus*, and *Streptococcus sanguinis*, salivary enzymes like lysozyme and lactoperoxidase are crucial for maintaining oral health. A protein with special enzymatic activity called salivary lactoperoxidase (LPO) shields salivary proteins from bacterial deterioration. It functions in tandem with hydrogen peroxide and thiocyanate. The end products of this interaction oxidise bacterial sulfhydryl groups and prevent the metabolism of glucose [13].

Complex interactions between the host and microbial elements in the oral cavity led to dental caries. Saliva is a crucial model for examining the pathophysiology of dental caries because it is a medium that reflects both host and microbial dynamics and is essential to maintaining the balance of the oral environment [14]. Additionally, non-invasiveness, simplicity, high specificity, and high sensitivity are benefits of saliva-based testing that particularly make research on young children easier [15]. The role of salivary microbiome variation in dental caries development has been explained by a number of prior studies. However, there is still a lack of research on the salivary microenvironment’s function in the aetiology of ECC, particularly with regard to host factor features.

Saliva testing has been shown to have clinical validity and convenience [16]. However, children may experience anxiety and fear when using the dental explorer, which is used to collect plaque [17]. The few published studies on the total oral mycobiome and ECC to date have either used dental plaque or oral swab samples or exclusively focused on a fungal community without mentioning mouth bacteria. ECC, which manifests as one or more decaying, missing, or filled teeth or surfaces in the primary tooth, is the most prevalent disease in children globally [17]. Co-infection of *S. mutans* and *Candida albicans*, along with poor oral hygiene, genetic factors, and immunological factors, has been shown to cause ECC and dysbiosis of the oral microbiome.

The convergence of topic modelling, keyword co-occurrence, and pathway enrichment on a “Candida–Cytokine–Neuro” motif is particularly notable. Our text-level analyses show that Candida albicans, IL-6/TNF, and cortisol or related stress markers increasingly co-occur in abstracts discussing ECC and salivary dysbiosis. While co-mention cannot establish causation, these patterns support a testable hypothesis that heightened neuroendocrine stress and inflammatory signalling may create a permissive environment for fungal overgrowth and redox imbalance in susceptible children, which future prospective ECC cohorts can investigate.

Dental caries (DC) is an infectious, persistent, non-transmissible disease that is common worldwide [18]. According to estimates, this pathogenic condition affects about 2.8 billion people globally. The World Health Organisation (WHO) reports that the percentage of untreated DC cases in children’s first dentition is 42.7%, but in adults’ permanent dentition, it is 28.7% [19]. As a result, it is now acknowledged as a public health issue, with a high financial and health cost that mostly impacts the most vulnerable populations, such as low-income members of racial or ethnic minorities.

Numerous studies on DC have reported variations in the concentrations of these inflammatory mediators, especially in the saliva of children and young adults with moderate to severe carious lesions (exposure group) compared with those without caries or with mild caries (control group). This suggests that these cytokines may be involved in the pathophysiology of the disease and could be further studied as potential biomarkers for the diagnosis and follow-up of DC [20]. As a result, more research on this subject is warranted. Lastly, it is crucial to note that if DC is not treated promptly, it advances and invades the pulp tissue, resulting in discomfort and the development of granulomas and dental abscesses [21].

Both adults and children use cortisol, a hormone generated by the hypothalamic–pituitary–adrenal axis, as a stress indicator. Salivary cortisol levels are regarded as a non-invasive, accurate, and trustworthy way to quantify stress [22]. Salivary cortisol levels have been used to measure the impact of stress in several contexts, such as anxiety related to dental care, periodontal disease, and dental caries [23].

The Caruso et al. study’s findings demonstrated a connection between children’s caries experiences and salivary cortisol levels. Increased salivary cortisol levels, plaque, high tartar levels, and advanced age were all positively correlated with dental caries. Furthermore, the occurrence of caries was independently linked to both older age and elevated cortisol levels [23].

The multifunctional cytokine interleukin-6 (IL-6) is implicated in haematopoiesis, inflammation, and immunological response [24]. In reaction to infections and tissue damage, a variety of cell types, such as macrophages, fibroblasts, and endothelial cells, create it. IL-6 is a crucial biomarker in medical diagnostics since elevated levels are linked to a variety of inflammatory illnesses and disorders [25]. IL-6 levels in saliva may indicate the degree and activity of the disease and reflect the underlying inflammatory processes, especially in ECC and RC, where tissue degradation happens quickly [24,25,26,27].

According to a study by Padmanabhan et al., children with ECC and RC had higher salivary IL-6 levels. Additionally, this study discovered that when the intensity and quantity of dental caries activity in these kids increase, so do their salivary IL-6 levels [28].

Both the mother and the child with S-ECC had high levels of *Candida albicans* infection, and the majority of the fungal strains were genetically related. This suggests that the mother may be the main source of *Candida albicans* acquisition in the oral cavity of children with S-ECC. Additionally, a high association between the carriage of *S. mutans* and *Candida albicans* in saliva and plaque was found, indicating a symbiotic relationship between the two bacteria in the context of ECC [29].

Alarcón-Sánchez et al.’s meta-analysis revealed a notable rise in TNF-α and IL-6 levels. These cytokines might be useful as supplementary indicators for determining how severe DC is [30].

This integrative work connects computational patterns drawn from literature with biological plausibility, revealing a complex development of ECC–saliva research. The subject innovation landscape shows how the focus of research has shifted from fluoride efficacy to salivary neuro-immune signalling and host response biomarkers. Knowledge graphs identify the mechanistic relationship between oxidative stress and cytokine activation, whereas Prophet-based predictions show a sustained increase in antioxidant and proteomic research. Crucially, Hypothesis 3 provides a conceptual framework for upcoming translational research connecting stress, immunity, and microbial ecology in ECC by consolidating data for the *Candida*–Cytokine–Neuro axisCorrect.

### Limitations

This study has several important limitations. First, we analysed only PubMed titles and abstracts; we did not mine full texts or other databases such as Scopus or Web of Science, so the corpus may be incomplete and is sensitive to how authors frame their work. Second, text-based co-occurrence and topic proximity reflect how concepts are discussed together, not underlying biological interactions; they cannot distinguish correlation from causation. Third, our Novelty Index and community structures are internally constructed exploratory metrics rather than established bibliometric standards, and we use them only for descriptive ranking. Fourth, the illustrative neuroimmune dataset is entirely synthetic and was created solely to demonstrate a potential analytic pipeline for future ECC cohorts; its confusion matrix and SHAP values should not be interpreted as clinical performance estimates. Finally, our results may be influenced by “spin” in abstracts, where benefits are overstated relative to full-text findings, highlighting the need for follow-up systematic reviews and prospective studies focused on the candidate neuro-immunometabolic axes identified here.

## 5. Conclusions

Early childhood caries (ECC) is a highly prevalent, multifactorial disease, and the ECC–saliva literature mirrors this complexity. By treating PubMed titles and abstracts as data, our AI-assisted pipeline mapped seven coherent thematic clusters and highlighted antioxidant/redox, proteomic, peptide-immunity, and Candida-related topics as comparatively underexplored ECC–saliva domains. The emergent Candida–cytokine–neuroendocrine motif and the emphasis on oxidative stress and salivary defence peptides do not establish causality, but they provide a structured, literature-grounded set of hypotheses that can be prioritised in future clinical and mechanistic studies. In keeping with trends towards more holistic, host-centred paediatric dentistry, this work demonstrates how bibliometric topic modelling, network analysis, and temporal forecasting can be harnessed to design next-generation ECC cohorts focused on neuro-immunometabolic salivary markers.

## Figures and Tables

**Table 1:** BERTopic-Derived Topic Clusters.

Topic	Representative Keywords	Count
0	Carie, Child, Ecc, Group, Salivary	216
1	Carie, Risk, Child, Ecc, Fluoride	72
2	Protein, Carie, Salivary, Antioxidant	33
3	Candida, Albican, Oral, Child, Biofilm	25
4	Fluoride, Remineralization, Enamel	25
5	Peptide, Immunity, Lactoferrin, Defensin	23
6	Tac, Antioxidant, Gpx, Sod, Saliva	18

**Table 2:** Topic clustering and representative keywords (BERTopic summary).

Topic	Count	Name	Top Keywords	Representative Abstracts
−1	10	Unclassified	carie, child, group, salivary, ecc, use, early, microbiome	Comparative assessment of antimicrobial activity…
0	216	Caries-Child-ECC-Group	caries, child, ecc, group, salivary, early, childhood, prevalence	Comparative evaluation of salivary trefoil factor…
1	72	Caries-Risk-ECC-Fluoride	carie, risk, child, ecc, fluoride, enamel, tooth, remineralization	Fluoride varnish applications and ECC prevention…
2	33	Protein-Caries-Salivary-Antioxidant	protein, saliva, antioxidant, defence, oxidative, immune	Proteomic analysis of antioxidant proteins in ECC…
3	25	Candida-Child-Biofilm	candida, albicans, oral, biofilm, fungal, microbe, carie	Association of oral *Candida albicans* with severe ECC…
4	25	Fluoride-Remineralization-Enamel	fluoride, enamel, remineralization, tooth, protective	Effect of nano-hydroxyapatite on enamel repair…
5	23	Peptide-Immunity-Defensin-Lactoferrin	peptide, defensin, lactoferrin, innate, mucosal, immunity	Salivary antimicrobial peptides as ECC risk markers…
6	18	TAC-Antioxidant-Carie-Child	tac, antioxidant, gpx, sod, redox, oxidative, stress, saliva	Antioxidant enzyme activities in ECC-affected saliva…

**Table 3:** Topic novelty, density, and diversity summary.

Topic ID	Documents (*n*)	Simplified Topic Name	Density Score	Diversity Score	Novelty Index (Higher the Number = More Novel)
6	18	TAC-Antioxidant-Child	0.077	0.0033	0.043
5	23	Protein-Salivary-ECC	0.098	0.0030	0.031
4	25	Fluoride-Tooth-Group	0.107	0.0030	0.028
3	25	Child-Caries-ECC-Mother	0.107	0.0027	0.025
2	33	Salivary-Caries-Level	0.141	0.0033	0.024
1	37	Caries-Risk-ECC-Child	0.158	0.0027	0.017
0	73	Caries-Oral-ECC-Child	0.312	0.0033	0.011

**Table 4:** Temporal trend forecast (Prophet Analysis).

Topic ID	Simplified Name	Historical Trend (2000–2024)	Forecast (2025–2030)	Growth Rate (%/yr)	Trend Type
0	ECC-Oral-Epidemiology	Plateau since 2020	Slight decline projected	−1.5	Stable/Saturated
1	ECC-Risk-Child-Fluoride	Steady 2005–2020	Moderate rise till 2027	+2.3	Gradual Increase
2	Protein-Salivary-Antioxidant	Sparse until 2015	Rapid growth expected	+8.7	Exponential Rise
3	Candida-Biofilm-Infection	Irregular before 2018	Mild growth, stabilising	+3.1	Stabilising
4	Fluoride-Enamel- Remineralization	Mature topic	Flat projection	0.0	Saturated
5	Peptide-Immunity-Lactoferrin	Emerging after 2018	Sustained high growth	+9.8	Accelerating
6	TAC-Antioxidant-Redox	Rare before 2020	Rapid surge projected	+11.4	Emerging Hotspot

**Table 5:** Top 10 journals publishing ECC–saliva articles in the corpus.

Journal	Number of Articles
*International Journal of Clinical Paediatric Dentistry*	20
*The Journal of Clinical Paediatric Dentistry*	17
*BMC Oral Health*	15
*Journal of the Indian Society of Pedodontics and Preventive Dentistry*	11
*International Journal of Paediatric Dentistry*	10
*European Archives of Paediatric Dentistry: Official Journal of the European Academy of Paediatric Dentistry*	9
*Archives of Oral Biology*	9
*Caries Research*	9
*European Journal of Paediatric Dentistry*	8
*Frontiers in Cellular and Infection Microbiology*	8

**Table 6:** Observed and forecasted publication trends per topic.

Topic ID	Topic Label	Last Observed Year	Last Observed Count	Predicted 2026 Count	Predicted 2029 Count	Forecast Slope 2026–2029
−1	Noise/miscellaneous	2025	5	4.93	5.44	0.169
0	Candida/oral microbiome	2025	3	7.79	8.82	0.342
1	ECC risk factors and epidemiology	2025	3	2.89	3.19	0.100
2	General salivary biomarkers	2025	2	3.08	3.45	0.124
3	Maternal–child ECC and behaviours	2020	2	0.52	0.43	−0.030
4	Fluoride and tooth-level prevention	2025	5	1.95	2.15	0.068
5	Salivary proteins and peptides	2025	2	2.33	2.62	0.099
6	Antioxidant/TAC salivary defence	2024	3	1.69	1.90	0.069

## Data Availability

The ECC–saliva PubMed corpus underlying this work was retrieved using the query (“early childhood caries” AND saliva) on 29 October 2025. Processed text features, topic assignments, and derived indices (e.g., novelty scores and keyword networks) will be made available in a public repository together with all Python scripts used for text cleaning, topic modelling, network analysis, and forecasting upon acceptance of the manuscript. The ECC_NeuroImmune_integrated_pilot.csv dataset distributed with this repository is purely synthetic and is provided only as a demonstration of the proposed modelling pipeline; it contains no real patient data.

## Abbreviations

The following abbreviations are used in this manuscript:

ECC	Early childhood caries
S-ECC	Severe early childhood caries
CDCP	The Center for Disease Control and Prevention
NI	Novelty index
LPO	Lactoperoxidase
DC	Dental caries
WHO	The World Health Organisation
